# Efficacy of an oral combination of afoxolaner and milbemycin oxime for the prevention of transmission of *Babesia canis* by *Dermacentor reticulatus* ticks to dogs

**DOI:** 10.1186/s13071-025-06787-y

**Published:** 2025-04-15

**Authors:** Eric Tielemans, Carin Rautenbach, Alta Viljoen, Frederic Beugnet

**Affiliations:** 1 Boehringer Ingelheim Animal Health, Lyon, France; 2 Clinvet International (Pty) Ltd., Bloemfontein, Republic of South Africa

**Keywords:** Afoxolaner, Dog, *Babesia canis*, *Dermacentor reticulatus*, Prevention of transmission

## Abstract

**Background:**

Canine babesiosis is a tick-borne disease of significant veterinary importance in dogs. It is caused by *Babesia canis* in Europe, where it is transmitted by *Dermacentor reticulatus* ticks.

**Methods:**

A blinded, randomized, good clinical practice (GCP) and negative control experimental study was conducted to verify the efficacy of NexGard Spectra® in reducing the transmission of *B. canis* by *D. reticulatus* to dogs. NexGard Spectra® (IVP) is an oral product for dogs combining afoxolaner, an acaricide/insecticide compound from the isoxazoline class, and milbemycin oxime, a nematicide compound from the macrocyclic lactone class. Three groups of eight dogs were used; one group orally treated on day 0 with the IVP at the minimum recommended dose and two untreated control groups. On day 1, dogs from the treated group and from control group 1 were infested with 50 *D. reticulatus* adult ticks of 50/50 sex ratio infected with *B. canis* at a 23% infection rate. On day 28, dogs from the treated group and from control group 2 were infested similarly to those on day 1. Ticks were removed 6 days after each infestation.

**Results:**

Seven to nine days after each infestation, all untreated control dogs displayed clinical signs of canine babesiosis, i.e., lethargy, and/or dark urine, and/or > 39.5 °C rectal temperature. Blood was collected for microscopical blood smear examination, and for polymerase chain reaction (PCR) analysis. The blood smears from all untreated control dogs were positive for *Babesia* and all the PCR analyses were positive for *B. canis.* The control dogs were rescue treated. All control dogs were confirmed positive for *B. canis* by IFA on day 21 (control group 1) and on day 42 (control group 2). None of the IVP-treated dogs expressed any clinical sign of canine babesiosis following each of the two infestations of days 1 and 28 and until day 56. Blood was collected for IFA and PCR analyses from the treated dogs on days 21, 28, 42, and 56, and all results were negative.

**Conclusions:**

In this study, the antiparasitic treatment prevented the transmission of *B. canis* to dogs following induced infestations.

**Graphical Abstract:**

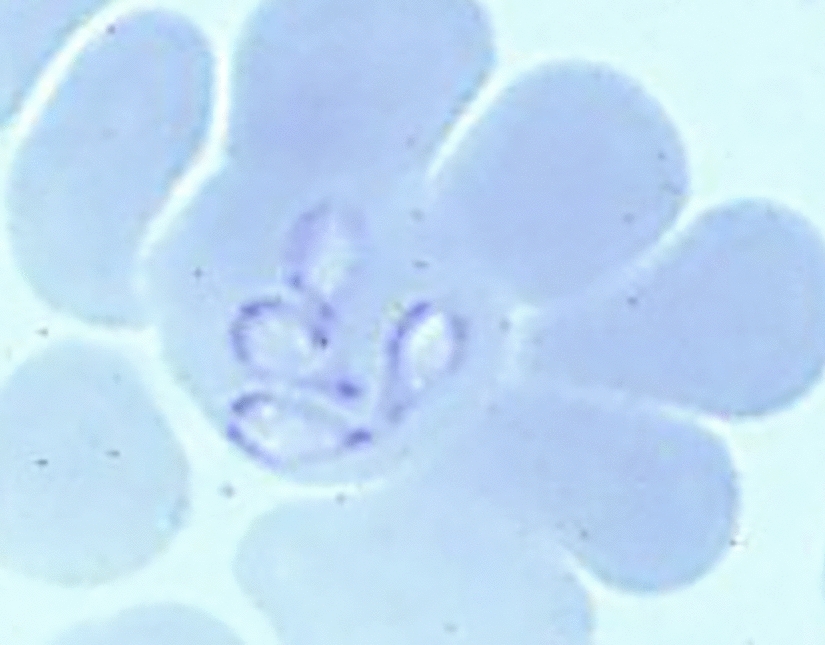

## Background

Blood parasites of the genus *Babesia* are mainly transmitted by ixodid ticks (family Ixodidae) to a broad range of mammalian species, including domestic and wild animals, as well as humans [1–5]. *Babesia* spp. have a significant medical and economic importance and are considered the second most common blood parasite of mammals after trypanosomes [6]. *Babesia* species occur worldwide, where most species appear to be geographically constrained by the distribution of their reservoir hosts and vectors [7]. The pathological effect of *Babesia* spp. in the vertebrate host is caused by the invasion and destruction of erythrocytes in which sporozoites undergo asexual reproduction by binary fission, resulting in a replicative cycle with multiplied infective merozoites. Consequently, *Babesia* infections commonly cause systemic inflammatory reactions and hemolysis-related symptoms such as febrile syndrome, anemia, hemoglobinuria, jaundice, lethargy, and organ failure in the longer term. The severity of babesiosis can vary in intensity, depending on the host’s inherent conditions (genetic factors, age, concurrent infection with other pathogens, immunological status) and the infecting *Babesia* species [8–10].

Canine babesiosis is a disease of major veterinary importance, affecting dogs worldwide. Several identified canine *Babesia* species are variable in term of geographical distribution, vector, severity, clinical signs, and treatment sensitivity [3, 8, 9]. Large-form species, e.g., *B. canis* and *B. vogeli* (and *B. rossi* as imported cases) and small-form species, e.g., *B. vulpes* and *B. gibsoni*, are reported in Europe [11].

*Babesia canis* is described across most of Europe, especially in wet and temperate-to-cold central regions, where *Dermacentor reticulatus*, its recognized vector, thrives [12]. Canine babesiosis caused by *B. canis* is usually characterized by acute onset. It causes moderate-to-severe clinical symptoms, often associated with hemolytic anemia and thrombocytopenia, sometimes with poor prognosis [9, 10].

NexGard Spectra® is an endectoparasiticide oral product for dogs, combining afoxolaner and milbemycin oxime. Afoxolaner is an insecticide and acaricide of the isoxazoline class. Isoxazolines antagonize the GABA-gated chloride ion channels of the hematophagous arthropods that feed on the treated host. Milbemycin oxime, the other active ingredient of the product, is a nematicide of the macrocyclic lactone class, with negligible efficacy on arthropods (unpublished data). The effectiveness of afoxolaner for the prevention of *B. canis* experimentally transmitted by *Dermacentor reticulatus* was already demonstrated in an experimental study with oral afoxolaner administered in a mono-product (NexGard®), at label dose, and in comparison with a single untreated control group [13]. This manuscript describes a new study, designed according to the most recent relevant guideline for studies evaluating the efficacy of parasiticides in reducing the risk of vector-borne pathogen transmission [14], namely requiring treatment at the minimum recommended dose, infective challenges repeated at the beginning and at the end of efficacy claim duration, and with a distinct control group for these two challenges.

## Methods

### Study design

This study, conducted in 2024, was designed in accordance with good clinical practices as described in International Cooperation on Harmonization of Technical Requirements for Registration of Veterinary Medicinal Products (VICH) guideline GL9, and was designed in accordance with the World Association for the Advancement of Veterinary Parasitology (W.A.A.V.P.) guidelines for studies evaluating the efficacy of parasiticides in reducing the risk of vector-borne pathogen transmission in dogs and cats [14].

This study was conducted under a blinded and randomized design.

The efficacy assessment was based on a comparison of *Babesia* infection level in a treated group and untreated control groups following infestations with *D. reticulatus* infected with *B. canis*, 1 day and 28 days after treatment.

The study outline and most animal characteristics per study group are presented in Table 1.

**Table 1:** Animal characteristics and study outline

Groups		Animal characteristics at inclusion (on Day −2)			IVP treatment	*D. reticulatus*		Daily rectal temperatures^1^	PCR and IFA analyses^2^
	*n*	Sex	Age (months)	Body weight (kg)		Infestation(s)	Removal(s)		
Control 1	8	4 m, 4 f	19–88	12.1–20.0	NA	Day 1	Day 7	Days 1–56	Days 21 and 28
Control 2	8	3 m, 5 f	31–88	12.9–23.4	NA	Day 28	Day 34	Days 1–56	Days 42 and 56
IVP	8	4 m, 4 f	28–82	14.2–23.8	Day 0	Days 1 and 28	Days 7 and 34	Days 1–56	Days 21, 28, 42, and 56

Animals were examined for clinical signs once a day until day 6 and twice daily from day 7 to day 56

^1^ Whenever the rectal temperature exceeded 39.5 °C, and/or babesiosis was suspected on the basis of clinical signs, blood was collected for blood smear microscopical examination and PCR analysis. A dog with a positive blood smear was rescue treated

^2^ A dog was removed from the study once a PCR and an IFA analysis were positive

### Animals and husbandry

A total of 24 healthy purpose-bred adult dogs (14 Beagles, four beagle-crosses, and six mongrels), 11 males and 13 females, aged 19–88 months, and weighing 12.1–23.8 kg were included in the study.

The containment of the dogs complied with the South African National Standard SANS 10386: latest version “The care and use of animals for scientific purposes.” The animals were kept individually in cages and had visual and auditory contact with conspecifics. Dogs were socialized weekly. The cages had concrete floors to facilitate cleaning and each cage was fitted with a sleeping bench. At least one toy/chew was made available to each dog and was replaced weekly. The dog cages were part of an environmentally controlled indoor animal unit; temperatures ranged between 16 °C and 25 °C. A photoperiod of 12 h light–12 h darkness was maintained. The floor size of each dog cage was 3.0 m × 1.9 m. No substances with an insecticidal or acaricidal activity were used on the animals or in their environment. Standard commercially available diets (VetsBrands Premium adult maintenance dog food or Hill’s Adult Large Breed Dog Food) that met the nutritional requirements of the animals were provided at the recommended rates. Any dog diagnosed positive for *Babesia* was immediately rescue treated and provided Hill’s a/d for 3 days. Potable water, as supplied by the local municipality, was provided in stainless steel bowls and replenished at least twice daily.

### *Dermacentor reticulatus* and *B. canis* isolates

The *D. reticulatus* ticks used in this study were originally obtained from BLE Laboratories (Ireland) in 2007. They had been genetically enriched with *D. reticulatus,* obtained from the Utrecht Centre for Tick-borne Diseases (UCTD), Netherlands, in 2009, 2012, 2014, and 2017.

The *B. canis* parasites used in this study originated from the Netherlands. A laboratory-bred dog was inoculated with blood derived from a clinical case of canine babesiosis, and blood from this dog served as infectious material for the ticks used in this study. A batch of *D. reticulatus* nymphs were fed on the infected dog. The adult ticks obtained from the engorged nymphs were infected at a 34% rate, as determined by polymerase chain reaction (PCR) on a sample of 50 ticks.

### Pretreatment activities

In total, 30 dogs were acclimated to study conditions since day −7. A clinical examination was performed on all dogs on day −7. To evaluate their suitability for tick infestation, all dogs were infested on day −7, following the method described in the tick infestation and removals paragraph, with the exceptions that the *D. reticulatus* were uninfected and were removed and counted 2 days after infestation, on day −5. On day −5, blood was collected from all dogs for immunofluorescence assay (IFA) and PCR analyses to ensure they were not positive for *B. canis* before inclusion. The 24 dogs with the highest live attached tick count and that were suitable for the study, as determined by the investigator, were selected for inclusion. Eight blocks with three dogs each were defined on the basis of live attached tick numbers, allowing a randomization to three groups of eight dogs each, one treated group and two control groups. The numbers of live attached ticks in the included dogs ranged from 13 to 35, and the average numbers of ticks were 22.9, 23.0, and 22.9 in the control groups 1 and 2 and the IVP-treated group, respectively.

### Treatment

On day 0, dogs from the treated group were orally administered a combination of whole tablets defined according to their body weight and to target the minimum label dose of the product, i.e., 2.5 mg/kg afoxolaner and 0.5 mg/kg milbemycin oxime, as described in Table 2. The mean administered afoxolaner dose was 2.75 mg/kg.

**Table 2:** Individual treatment on day 0

Dog		Tablet combination*	Dose (mg)		Dosage (mg/kg)	
#	Body weight (kg)		Afoxolaner	MO	Afoxolaner	MO
1	15.95	9/2 + 38/8	46.875	9.375	2.94	0.59
2	21.55	19/4 + 38/8	56.250	11.250	2.61	0.52
3	18.75	19/4 + 38/8	56.250	11.250	3.00	0.60
4	18.45	9/2 + 38/8	46.875	9.375	2.54	0.51
5	19.65	19/4 + 38/8	56.250	11.250	2.86	0.57
6	14.15	38/8	37.500	7.500	2.65	0.53
7	23.80	9/2 + 19/4 + 38/8	65.625	13.125	2.76	0.55
8	14.35	38/8	37.500	7.500	2.61	0.52
				***Mean***	***2.75***	***0.55***

^*^NexGard Spectra® strengths:

9/2 = 9.375 mg afoxolaner & 1.875 mg milbemycin oxime (for dogs 2–3.5 kg)

19/4: 18.75 mg afoxolaner & 3.75 mg milbemycin oxime (for dogs > 3.5–7.5 kg)

38/8: 37.5 mg afoxolaner & 7.5 mg milbemycin oxime (for dogs > 7.5–15 kg)

### Tick infestations and removals

The treated group was infested on days 1 and 28. Two control groups were used to verify the adequacy of each *B. canis* transmission and were thus infested on day 1 (control group 1) or day 28 (control group 2). Each time, dogs were infested with 50 (± 2) adult ticks of 50/50 sex ratio. To obtain 50 ticks with a consistent *B. canis* infection rate for each individual infestation, 34 ticks from the infected batch (17 males and 17 females) with a 34% infection rate as determined by PCR, were combined with 16 ticks (8 males and 8 females) from the same origin, but that had not been exposed to *B. canis* infected blood. Therefore, the *B. canis* infection rate for each batch of 50 ticks used for infestations was 23%. To facilitate tick infestation, dogs were sedated and placed in an infestation crate for 1–2 h. The ticks were released on the back or flank of the animal and were allowed to disperse into the hair coat.

Then, 6 days after each infestation, on days 7 and 34, the remaining ticks were removed and counted from each infested group (the treated group and control group 1 on day 7, and the treated group and control group 2 on day 34).

### Health and *Babesia* infection evaluations

General health observations were performed once daily from day −7 to day 6 and twice daily from day 7 to day 56. Clinical examinations were performed weekly from day 7 to day 56. Rectal temperature was measured once daily from day 1 to day 56.

Whenever the rectal temperature exceeded 39.5 °C and/or babesiosis was suspected on the basis of clinical signs (e.g., lethargy, dark urine, diarrhea, anorexia), blood was collected for blood smear processing and microscopic search of *Babesia* in red blood cells, and for *B. canis* PCR analysis, recognized methods for babesia diagnosis [15]. Irrespective of clinical signs, blood collection was scheduled for *B. canis* IFA and PCR analyses on days 21 and 28 (treated group and control group 1), and on days 42 and 56 (all dogs).

For *B. canis* PCR analysis, total genomic DNA was isolated from whole blood samples using a commercial genomic DNA isolation kit (GeneJET Genomic DNA Purification Kit, Thermo Fisher Scientific, USA). PCR entailed using primers specific to the 18S Ribosomal DNA region of *B. canis*, and as referenced [16]. The PCR procedure followed a recognized method [17]. For IFA analysis, serum was collected from blood tubes and was assayed for *B. canis* antibodies using a commercially available IFA test kit (MegaFLUO® BABESIA canis, Megacor Diagnostik, Austria).

### Statistical analysis

The primary efficacy criterion was the number of dogs that became infected with *B. canis* (when tested positive for *B. canis* by both PCR analysis and IFA serology). The infection rate of dogs in groups that became infected during the study were summarized descriptively. The percentage blocking efficacy for the IVP-treated group was calculated as follows in relation to the day 1 and day 28 infestations:

$${\text{Blocking}}\;{\text{efficacy}}\left( \% \right) = {1}00 \times \left( {{\text{Tc}} - {\text{Tt}}} \right)/{\text{Tc}}$$

Tc is the total number of untreated control dogs that became infected (control group 1 for day 1, control group 2 for day 28); Tt is the total number of dogs that became infected in the IVP group (group 3).

SAS Version 9.4 was used for all the statistical analyses.

### Endpoints

Whenever a dog was diagnosed positive for *Babesia* infection on a blood smear, blood for PCR analysis was collected, and the dog was rescue treated. All study activities for rescue treated dogs were stopped except the scheduled blood collections for IFA and PCR analyses. A rescue-treated dog was removed from the study once a positive IFA result was obtained.

## Results

The individual results of tick counts and *Babesia* infection diagnosis are described in Table 3.

**Table 3:** Individual results of tick counts and *Babesia* diagnosis

Group^1^	#	Live (dead) attached tick count^2^		Original diagnosis of canine babesiosis				Scheduled bioanalyses of *Babesia canis*							
				Day of diagnosis, clinical signs of babesiosis, and/or rectal temperature of > 39.5 °C		Blood smear	PCR	IFA				PCR			
		Day 7	Day 34					Day 21^3^	Day 28	Day 42^3^	Day 56	Day 21	Day 28	Day 42	Day 56
Control 1	1	25	–	Day 9:	Lethargic	Positive	Positive	Positive	–	–	–	Negative	–	–	–
Control 1	2	37	–	Day 9:	39.8 °C	Positive	Positive	Positive	–	–	–	Negative	–	–	–
Control 1	3	13	–	Day 9:	40.6 °C	Positive	Positive	Positive	–	–	–	Negative	–	–	–
Control 1	4	44	–	Day 8:	40.2 °C, lethargic	Positive	Positive	Positive	–	–	–	Negative	–	–	–
Control 1	5	41	–	Day 8:	40.0 °C, lethargic	Positive	Positive	Positive	–	–	–	Negative	–	–	–
Control 1	6	44	–	Day 8:	39.8 °C, lethargic	Positive	Positive	Positive	–	–	–	Negative	–	–	–
Control 1	7	42 (2)	–	Day 9:	40.1 °C	Positive	Positive	Positive	–	–	–	Negative	–	–	–
Control 1	8	42	–	Day 9:	39.9 °C, dark urine	Positive	Positive	Positive	–	–	–	Negative	–	–	–
Control 2	1	–	27	Day 36:	Lethargic	Positive	Positive	–	–	Positive	–	–	–	Negative	–
Control 2	2	–	29	Day 37:	Dark urine	Positive	Positive	–	–	Positive	–	–	–	Negative	–
Control 2	3	–	25	Day 35:	39.7 °C	Positive	Positive	–	–	Positive	–	–	–	Negative	–
Control 2	4	–	40	Day 36:	40.2 °C	Positive	Positive	–	–	Positive	–	–	–	Negative	–
Control 2	5	–	34	Day 35:	Lethargic	Positive	Positive	–	–	Positive	–	–	–	Negative	–
Control 2	6	–	23	Day 36:	Lethargic, dark urine	Positive	Positive	–	–	Positive	–	–	–	Negative	–
Control 2	7	–	26	Day 35:	39.6 °C	Positive	Positive	–	–	Positive	–	–	–	Negative	–
Control 2	8	–	39	Day 36:	40.0 °C	Positive	Positive	–	–	Positive	–	–	–	Negative	–
IVP	1	0	0	No abnormal sign or rectal temperature		–	–	Negative	Negative	Negative	Negative	Negative	Negative	Negative	Negative
IVP	2	0 (2)	0 (5)	No abnormal sign or rectal temperature		–	–	Negative	Negative	Negative	Negative	Negative	Negative	Negative	Negative
IVP	3	0 (3)	0 (3)	No abnormal sign or rectal temperature		–	–	Negative	Negative	Negative	Negative	Negative	Negative	Negative	Negative
IVP	4	0 (3)	0 (4)	No abnormal sign or rectal temperature		–	–	Negative	Negative	Negative	Negative	Negative	Negative	Negative	Negative
IVP	5	0 (1)	0 (2)	No abnormal sign or rectal temperature		–	–	Negative	Negative	Negative	Negative	Negative	Negative	Negative	Negative
IVP	6	0	0 (2)	No abnormal sign or rectal temperature		–	–	Negative	Negative	Negative	Negative	Negative	Negative	Negative	Negative
IVP	7	0 (5)	0 (1)	No abnormal sign or rectal temperature		–	–	Negative	Negative	Negative	Negative	Negative	Negative	Negative	Negative
IVP	8	0 (4)	0 (1)	No abnormal sign or rectal temperature		–	–	Negative	Negative	Negative	Negative	Negative	Negative	Negative	Negative

^1^Control 1 dogs were infested on day 1 with 50 (± 2) *D. reticulatus* with a *B. canis* infection rate of 23%

Control 2 dogs were infested on day 1 with 50 (± 2) *D. reticulatus* with a *B. canis* infection rate of 23%

Treated group: dogs were treated on day 0 with NexGard Spectra® at a mean afoxolaner dose of 2.75 mg/kg and were infested on days 1 and 28 with 50 (± 2) *D. reticulatus* with a *B. canis* infection rate of 23%

^2^This column describes the counts of live (and dead) attached ticks. Otherwise, no live free ticks were found, and three dead free ticks were found on day 7 and day 34, each time in the treated group

^3^All dogs from control group 1 and control group 2 were removed from the study on days 21 and 42, respectively, and no further evaluation was performed as they had been confirmed positive for *B. canis* by IFA analysis

The tick infestations on days 1 and 28 were demonstrated to be adequate by their corresponding control groups 1 and 2, as 6 days later, on days 7 and 34, respectively, a significant number of live attached ticks were found. The mean number of live attached ticks was 36 (72%) in the untreated control group 1 and 30.4 (60.8%) in the untreated control group 2. No live attached tick was found on any IVP treated dog on days 7 and 34, demonstrating a reduction of 100% of live attached ticks 6 days after infestation on days 1 and 28. A small number of dead attached ticks, ranging from 0 to 5, were found in this group.

A clear diagnosis of canine babesiosis was made for all 16 untreated control dogs, 7–9 days following their respective *D. reticulatus* infestation. All dogs expressed abnormal clinical signs, i.e., lethargy, and/or dark urine, and/or rectal temperature exceeding 39.5 °C. On the same day, the diagnosis of canine babesiosis was confirmed for each dog by a positive blood smear microscopical examination and *B. canis* PCR analysis. These 16 dogs were rescue treated on the day of diagnosis with injections of imidocarb dipropionate (7.2 mg/kg), diminazene aceturate (3.5 mg/kg) and several days of supportive medications, i.e., corticosteroid (prednisolone acetate 0.1 mg/kg), antiemetic (marbopitant 1 mg/kg), and hepatoprotectors. All dogs were confirmed positive by IFA analysis for *B. canis* on day 21 (control group 1) or day 42 (control group 2). The PCR analyses of these dogs on days 21 and 42 had turned negative, demonstrating the efficacy of rescue treatments. None of the IVP-treated dogs expressed any abnormal clinical signs of babesiosis, and their PCR and IFA bioanalyses performed on days 21, 28, 42, and 56 remained negative.

## Discussion

The data obtained in this study with the untreated animals demonstrated a reliable and consistent *B. canis* infection model and showed with the treated animals that afoxolaner completely blocked the *B. canis* transmission by *D. reticulatus* ticks.

*Dermacentor reticulatus* is a vector of numerous other protozoal, bacterial, and viral diseases [18], out of scope in this study, as the investigated model was limited to pathogen-free ticks experimentally infected only with *B. canis.*

Canine babesiosis is a highly significant and challenging disease. Several therapeutic or preventive veterinary strategies can be implemented alone or in combination. The protozoan can be directly targeted through pharmaceutical products or vaccinations. As described in this manuscript, the ixodid vector can be targeted through acaricidal products. The use of repellents can also reduce tick infestations.

Pharmaceutical treatment of dogs affected with canine babesiosis is challenging. A limited number of drugs used alone or in combination are available on- or off-label, with variable species and strain-related efficacy [11, 19, 20]. The mechanism of action of these drugs is mostly unclear. Injectable imidocarb dipropionate is registered for treating *B. canis,* nevertheless with cholinergic adverse effects and pain at injection. Intensive care is also necessary for severe manifestations of the disease, and relapses are possible. In this study, diminazene aceturate, a compound of poorly understood mechanism of action [21] and registered in South Africa for the treatment of *B. canis* in dogs, was used as treatment in combination with imidocarb dipropionate and with corticosteroid, hepato-protector, and antiemetic supportive medications.

Some vaccines containing soluble parasite antigens derived from in vitro cultures are registered against *B. canis* but with variable strain-related efficacy [22]. *Babesia* vaccines do not affect the infection but alter the disease progression and reduce the severity of the clinical signs. Research on recombinant antigens is currently underway and may provide new vaccines in the future [23, 24].

Repellent and acaricide products, such as permethrin and flumethrin, have been described as effective for the reduction of *B. canis* transmission [25–27], and more generally, for the prevention of several vector-borne diseases, including *Babesia* spp. [28].

The approach of blocking a pathogen transmission by using an acaricide targeting the vector has become an important veterinary strategy since the appearance of isoxazolines in 2014 [13, 29–33]. Contrarily to repellent products, isoxazolines do not prevent an arthropod vector from biting or attaching to its host and start a fluid exchange process. Nevertheless, *Babesia* spp. usually require several days after host attachment for the ookinetes to undergo several sporogony cycles, leading to the formation of infective sporozoites in the salivary gland of their ixodid vector [2, 12]. As examples, it was demonstrated in humans that *B. microti* was transmitted by *Ixodes scapularis* 36 h to 48 h after host attachment [34], or in cattle that *B. bigemina* was transmitted during the last 16 h to 24 h before vector (*Rhipicephalus microplus*) detachment [35]. Interestingly, in the present study, some dead attached *D. reticulatus* were found on the treated dogs but probably did not survive long enough to transmit their infective *B. canis* sporozoites.

Afoxolaner, alone or combined with milbemycin oxime, was demonstrated to be effective against *D. reticulatus* within 48 h of infestations and for 5 weeks after a treatment at minimum dose [36]. Afoxolaner was also effective in preventing *B. canis* transmission by *D. reticulatus* when administered at label dose in a mono-product (NexGard®) and with one control group verifying validity of weekly challenges [13]. The study described in this manuscript provides an upgraded design. It verified the efficacy of a single afoxolaner treatment administered at the minimum dose of its registered products and with a distinct control group verifying adequacy of infection after each challenge at the beginning (day 1) and end (day 28) of the efficacy claimed period. The 100% level of *B. canis* infection observed in both untreated control groups after challenges at day 1 or at day 28 supported the robustness of the model. The total absence of infection after the two infective tick challenges in the treated group supported the preventive efficacy of afoxolaner administered once and at minimum dose of its registered products.

## Conclusions

In this study, the killing effect of the investigated oral formulation combining afoxolaner and milbemycin oxime was fast and complete enough to interrupt the vector–host fluid exchange process, thus blocking tick capability to transmit the infectious *Babesia* sporozoites.

## Data Availability

No datasets were generated or analyzed during the current study.
